# Transcriptional heterogeneity of tumor-associated high endothelial venules defines inflammatory and stress-metabolic states with distinct prognostic associations

**DOI:** 10.1007/s12672-026-05162-2

**Published:** 2026-05-08

**Authors:** Hwal-Seok Choi, Yongjun Lee, Jin-Woo Choi, So-Jeong Kim, Hyunji Kim, Hyo-Kyung Pak, Jin Roh, Chan-Sik Park

**Affiliations:** 1 Department of Pathology, Asan Medical Center, University of Ulsan College of Medicine, Seoul, Republic of Korea; 2 Asan Institute for Life Science, Asan Medical Center, University of Ulsan College of Medicine, Seoul, Republic of Korea; 3 Department of Biochemistry and Molecular Biology, Asan Medical Center, University of Ulsan College of Medicine, Brain Korea 21 Project, Seoul, Republic of Korea; 4 Department of Pathology, Ajou University School of Medicine, Suwon, Republic of Korea

**Keywords:** High endothelial venules, Single-cell RNA sequencing, Transcriptomics, Prognosis

## Abstract

Tumor-associated high endothelial venules (TA-HEVs) mediate lymphocyte trafficking into tumors and modulate the tumor microenvironment, with reported effects on clinical outcomes. However, reports have described discordant associations across cancers and microenvironmental contexts. Studies on state-specific, pan-cancer analyses of TA-HEV function remain limited. We integrated publicly available single-cell RNA sequencing datasets from 11 cancer types. Functional features of TA-HEVs were inferred by pathway enrichment and single-cell gene-set scoring for pathway gene sets. State-specific programs were applied to The Cancer Genome Atlas dataset to assess their clinical impact. We constructed a comprehensive atlas of tumor-associated endothelial cells and identified TA-HEV subclusters. Five TA-HEV subclusters were grouped into two functional states: inflammatory and stress-metabolic. The inflammatory TA-HEVs were enriched for innate immune stimulation, cytokine/chemokine signaling, and MHC class II antigen presentation, whereas the stress-metabolic TA-HEVs were characterized by the unfolded protein response, heat shock pathways, oxidative phosphorylation, and ATP biosynthesis. Across cancers, the stress-metabolic TA-HEV state was generally associated with worse prognosis, while the inflammatory TA-HEV state showed context-dependent associations. Together, these findings define TA-HEVs as a heterogeneous endothelial population comprising distinct functional states with divergent clinical associations, providing a pan-cancer framework for interpreting TA-HEV signals in tumor biology.

**Supplementary Information:**

The online version contains supplementary material available at 10.1007/s12672-026-05162-2.

## Introduction

The tumor microenvironment plays a pivotal role in driving cancer progression, shaping therapeutic responses, and determining patient prognosis [1]. The tumor vasculature not only provides oxygen and nutrients but also serves as a critical gateway for regulating the entry of immune cells into tumor tissue [2]. In particular, high endothelial venules (HEVs) are specialized post-capillary venules that mediate lymphocyte trafficking into secondary lymphoid organs, and they have been observed across a variety of human cancers [3]. As evidence accumulated that HEVs within tumors may modulate the microenvironment through lymphocyte recruitment, studies of tumor-associated HEVs (TA-HEVs) have surged [4–7].

Early histological studies revealed that TA-HEVs are associated with favorable clinical outcomes across several cancer types, including breast cancer, gastric cancer, and head and neck cancers [2, 8, 9]. However, others have reported neutral or even adverse associations depending on tumor type, anatomic localization, and microenvironmental context [8, 10]. These discordant associations raise the possibility that TA-HEVs exist in distinct functional states shaped by local cues. Most prior studies have evaluated TA-HEVs within a single tumor type, with integrative efforts mostly limited to cross-study or meta-analytic approaches [10]. In addition, a systematic, state-resolved characterization of TA-HEVs across cancers has been lacking.

In the present study, we integrated publicly available single-cell RNA sequencing (scRNA-seq) datasets from 11 cancer types to construct a comprehensive atlas of tumor-associated endothelial cells (TA-ECs) and to identify TA-HEV populations, thereby enabling a state-resolved view of their transcriptional and functional heterogeneity.

## Results

### Transcriptional classification of tumor-associated endothelial cells

**Fig. 1:**
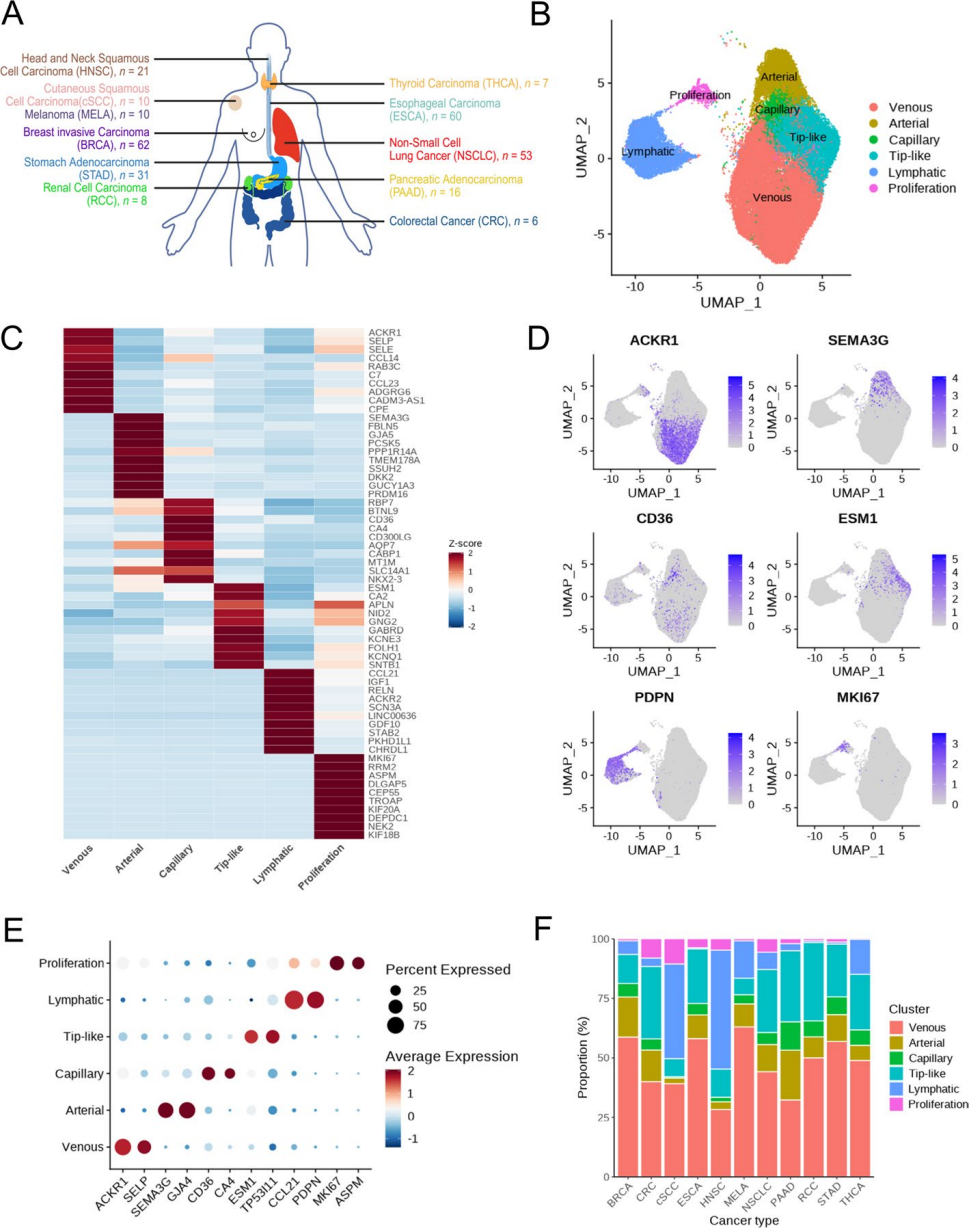
Single-cell transcriptomic clustering of endothelial cells (ECs) across multiple cancer types. **A** Overview of the study cohort showing the number of tumor samples, total single cells, and ECs included in the analysis across 11 cancer types. (Created with *BioRender.com*) **B** ECs were identified from the total single-cell population based on the expression of established EC markers (PECAM1, VWF, CDH5, PLVAP) and subjected to unsupervised clustering. **C** Each cluster was annotated as venous, arterial, capillary, tip-like, lymphatic, or proliferating ECs according to the expression patterns of top differentially expressed genes. Representative marker gene expression is shown in UMAP plots. **D** The expression of cluster-specific marker genes aligns with annotated cluster characteristics, confirming the accuracy and reliability of the clustering strategy. **E** Bubble plot showing the scaled expression and detection frequency of canonical marker genes across endothelial clusters. **F** The proportional distribution of each EC cluster across cancer types demonstrates that clusters are represented across diverse tumors without bias toward a specific tumor type

To reliably identify TA-HEVs across heterogeneous scRNA-seq datasets (Fig. 1A), we profiled 47,748 TA-ECs from 11 solid tumor types using canonical markers (PECAM1, VWF, CDH5, PLVAP). Unsupervised clustering revealed six EC subtypes (Fig. 1B)—venous, arterial, capillary, tip-like, lymphatic, and proliferative—defined by key differentially expressed genes (Supplementary Table S1): ACKR1 and SELP (venous), SEMA3G and GJA4 (arterial), CD36 and CA4 (capillary), ESM1 and TP53I11 (tip-like), CCL21 and PDPN (lymphatic), and MKI67 and ASPM (proliferative) (Fig. 1C-E). Venous ECs were the most abundant (50.7%), while proliferative ECs were rare (2.5%) (Fig. S1). At the tumor-type level, venous ECs were the dominant population in most cancers, whereas lymphatic and proliferative ECs were enriched in specific tumor contexts (Fig. 1F). These results indicated that TA-ECs can be clearly classified into six transcriptionally coherent subtypes, providing a transcriptional framework for the identification of the TA-HEV populations.

### Identification of TA-HEV subclusters within venous ECs

**Fig. 2:**
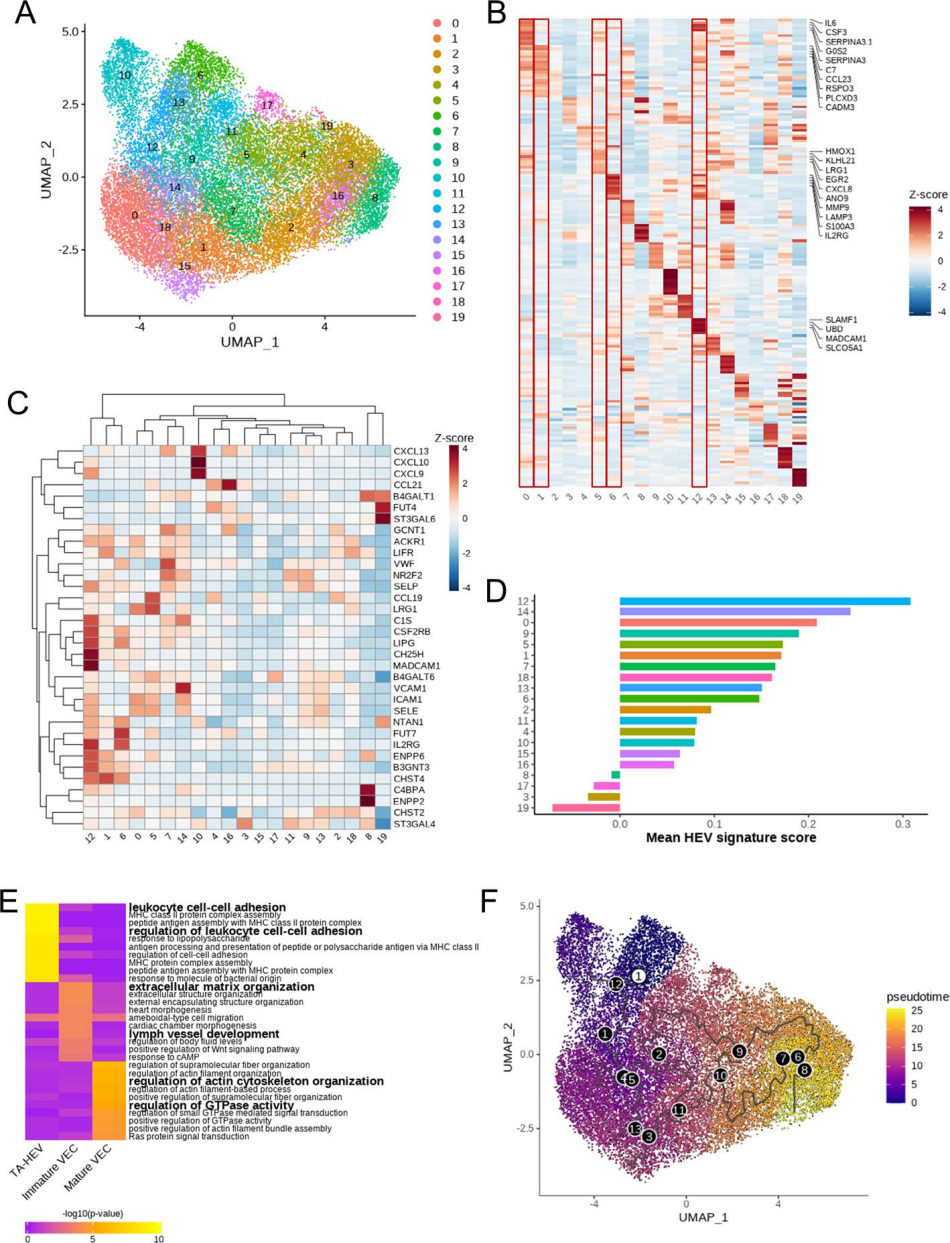
Identification of the tumor-associated high endothelial venule (TA-HEV) subcluster within the venous EC cluster. **A** To define the TA-HEV population, venous ECs identified in the initial clustering step were subjected to an additional round of unsupervised clustering, and the optimal resolution parameter was determined. **B** Heatmap showing the top differentially expressed genes across the 20 venous EC subcluster. Key HEV-associated marker genes are annotated; TA-HEV subclusters (0, 1, 5, 6, 12) are explicitly labeled to facilitate interpretation. **C** HEV gene expression patterns and **D** HEV signature scores across subclusters were analyzed to functionally classify venous ECs and to define the TA-HEV population. **E** Based on HEV gene expression and signature scores, venous ECs were divided into three compartments: TA-HEVs, immature venous ECs, and mature venous ECs. Pathway enrichment analysis demonstrated that each compartment exhibited coherent functional signatures, confirming the reliability of these annotations. **F** Pseudotime trajectory analysis revealed a continuum from immature to mature venous ECs, with TA-HEVs occupying a distinct region along the venous endothelial trajectory, consistent with their specialized endothelial identity

Next, we focused on venous ECs from the initial analysis to identify the TA-HEV EC population. Secondary unsupervised clustering identified 20 transcriptionally coherent subclusters (Fig. 2A–B and Supplementary Table S2). The top DEG heatmap (Fig. 2B) demonstrates that HEV-associated marker genes, including LRG1 and MADCAM1, are coherently co-enriched in the TA-HEV subclusters, with substantially reduced enrichment in the remaining venous EC subclusters. UMAP visualization showed that these subclusters were evenly distributed across tumor types without apparent dominance by a specific tumor type (Fig. S2). Based on HEV marker expression and HEV signature gene scores, five subclusters (0, 1, 5, 6, 12) were defined as classified as TA-HEVs, four (7, 9, 13, 14) as immature venous ECs, and the rest as mature venous ECs (Fig. 2C-D and Fig. S3). Pathway analysis based on differentially expressed genes (DEGs) of each venous subgroup showed that TA-HEVs were enriched in immune-related pathways, including leukocyte cell-cell adhesion (Fig. 2E and Supplementary Table S3). Immature ECs were enriched in developmental and extracellular matrix organization, and mature ECs were enriched in cytoskeletal regulation. These were consistent with their annotated biological identities. Pseudotime analysis revealed a trajectory from immature to mature ECs, with TA-HEVs occupying a coherent region along the venous endothelial trajectory (Fig. 2F), consistent with their annotation as a specialized venous endothelial state.

### Functional heterogeneity of TA-HEVs reveals inflammatory and stress-metabolic subtypes

**Fig. 3:**
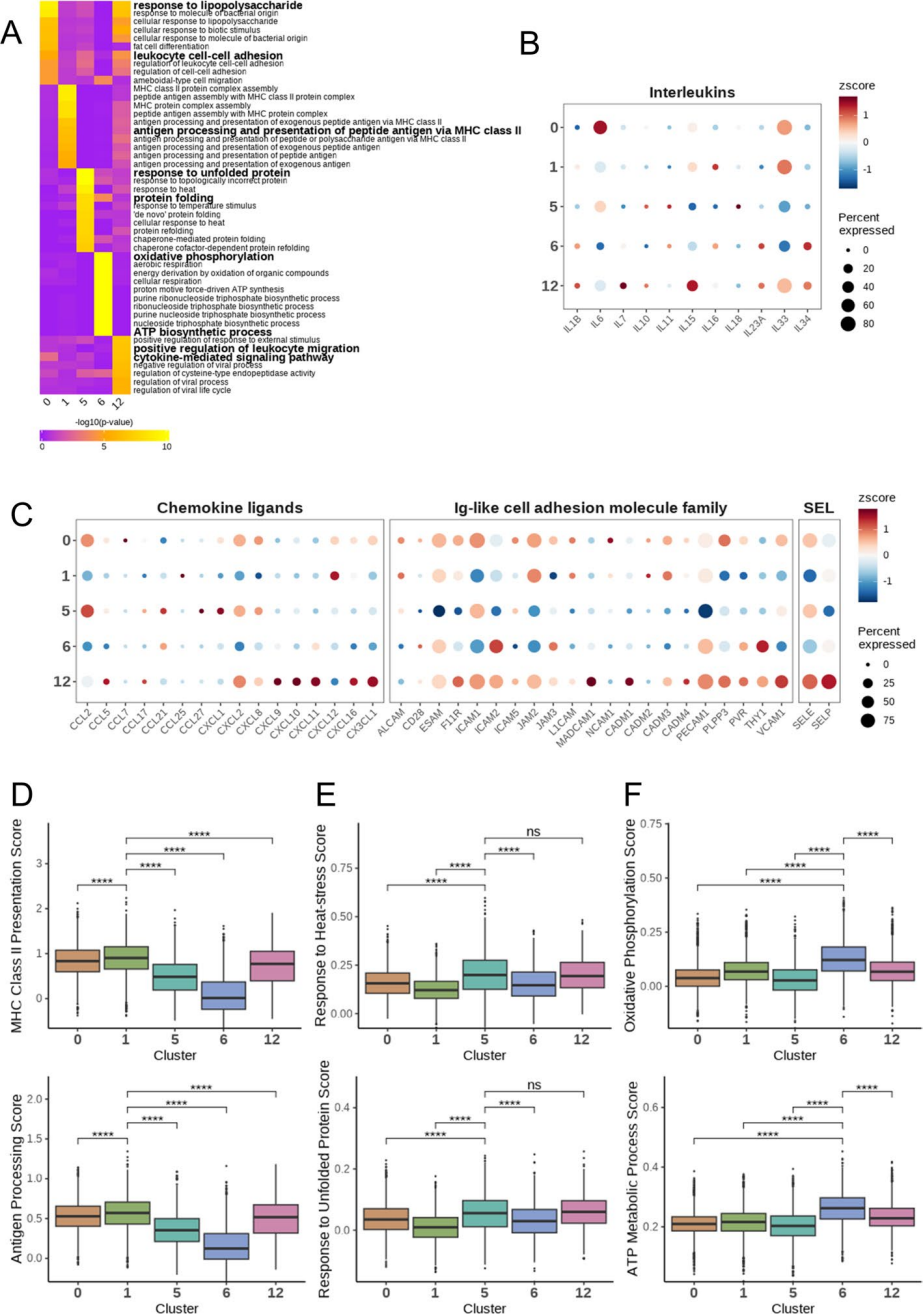
Functional heterogeneity of TA-HEV subclusters. Pathway enrichment analysis of the five TA-HEV subclusters. Subclusters 0, 1, and 12 were enriched for immune-related pathways, subcluster 5 for stress-related pathways, and subcluster 6 for metabolic processes. **B** Dot plots showing interleukin expression across TA-HEV subclusters, highlighting that immune-related subclusters exhibit elevated cytokine transcription. **C** Dot plots showing differential expression of chemokines, adhesion molecules, and selectins across subclusters, demonstrating cluster-specific regulation of leukocyte recruitment machinery. **D** Box plots of MHC class II and antigen-processing signature scores, showing that subcluster 1 is specialized for antigen presentation. **E** Box plots demonstrating selective enrichment of stress-related gene signatures in subcluster 5. **F** Box plots demonstrating enrichment of metabolic gene signatures in subcluster 6. Statistical significance was assessed using the Wilcoxon rank-sum test (*p < 0.05, **p < 0.01, ***p < 0.001, ****p < 0.0001)

Pathway analysis based on DEGs of the five TA-HEV subclusters defined above revealed two functional groups: inflammatory and stress-metabolic (non-inflammatory) (Fig. 3A and Supplementary Table S4). Among inflammatory TA-HEVs, subcluster 0 was enriched in pathways related to innate immune stimulation and leukocyte recruitment and expressed high IL6 (Fig. 3B and Fig. S4), suggesting early immune activation. Subcluster 12 was enriched in leukocyte migration and cytokine signaling, consistent with a role in immune activation and functional modulation (Fig. 3C). Subcluster 1 was enriched in MHC-II–mediated antigen presentation, supported by higher gene set scores for MHC-II (*p* < 0.001, *t*-test; Fig. 3D). Non-inflammatory TA-HEVs exhibited stress and metabolic signatures. Subcluster 5 showed higher gene set scores for endoplasmic reticulum (ER) and proteotoxic stress pathways, including heat stress and unfolded protein responses (*p* < *0.001* for both, *t*-test; Fig. 3E), and subcluster 6 showed higher gene set scores for oxidative phosphorylation and ATP biosynthesis (*p* < 0.001 for both, *t*-test; Fig. 3F). Thus, inflammatory TA-HEVs (0, 1, 12) are associated with immune-interactive transcriptional programs, while stress–metabolic TA-HEVs (5, 6) engage in stress adaptation and energy metabolism, demonstrating functional heterogeneity within TA-HEVs.

### Prognostic associations of TA-HEV states across cancer types

**Fig. 4:**
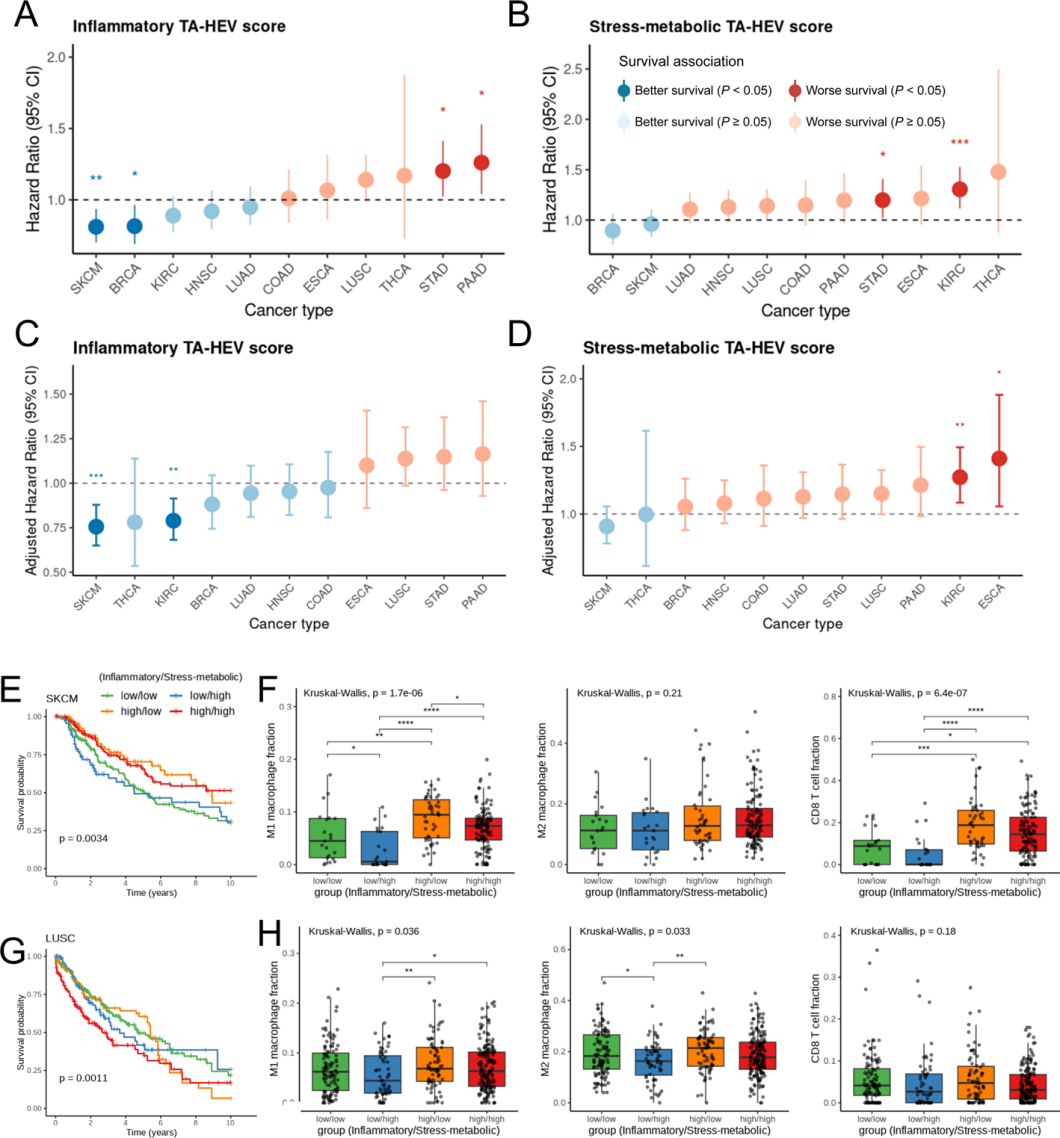
Clinical and immune-associated features of TA-HEV inflammatory and stress-metabolic states. **A–B** Forest plots of univariate Cox proportional hazards models using TA-HEV state scores in TCGA cohorts. **A** Inflammatory TA-HEV score. **B** Stress-metabolic TA-HEV score. Points show hazard ratios (HR) with 95% confidence interval (CI); colors denote direction (blue, HR < 1; red, HR > 1). **C–D** Forest plots of multivariate Cox proportional hazards models using TA-HEV state scores in TCGA cohorts. **C** Inflammatory TA-HEV score. **D** Stress-metabolic TA-HEV score. Points show hazard ratios (HR) with 95% confidence interval (CI); colors denote direction (blue, HR < 1; red, HR > 1). Multivariate models were adjusted for age, sex, and tumor stage. **E** Kaplan–Meier curves after stratifying patients into four groups by median splits of inflammatory and stress-metabolic scores (low/low, low/high, high/low, high/high) in SKCM; p values by log-rank test. **F** Kaplan–Meier curves after stratifying patients into four groups by median splits of inflammatory and stress-metabolic scores in LUSC; p values by log-rank test. **G** Box plots of selected immune fractions (e.g., M1/M2 macrophages, CD8⁺ T cells) across the four groups in SKCM. Group differences by Kruskal–Wallis test with post-hoc pairwise comparisons. **H** Box plots of selected immune fractions across the four groups in LUSC. Group differences by Kruskal–Wallis test with post-hoc pairwise comparisons. Statistical significance (*p < 0.05, **p < 0.01, ***p < 0.001, ****p < 0.0001). Abbreviations: BRCA, Breast invasive carcinoma; SKCM, Skin cutaneous melanoma; KIRC, Kidney renal clear cell carcinoma; LUAD, Lung adenocarcinoma; HNSC, Head and neck squamous cell carcinoma; COAD, Colon adenocarcinoma; THCA, Thyroid carcinoma; ESCA, Esophageal carcinoma; LUSC, Lung squamous cell carcinoma; STAD, Stomach adenocarcinoma; PAAD, Pancreatic adenocarcinoma

To evaluate the potential clinical relevance of TA-HEV functional heterogeneity, we generated subcluster-specific gene signatures and applied them to The Cancer Genome Atlas (TCGA) bulk RNA-seq data. Univariate and multivariate Cox regression analyses revealed cancer type-specific prognostic differences among TA-HEV subclusters (Fig. 4A–D). In univariate analysis (Fig. 4A, B), the inflammatory TA-HEV score was significantly associated with favorable survival in SKCM (HR = 0.81, 95% CI 0.70–0.94, *p* = 0.004) and BRCA (HR = 0.82, 95% CI 0.68–0.96, *p* = 0.017), but with worse outcomes in STAD (HR = 1.20, 95% CI 1.02–1.41, *p* = 0.027) and PAAD (HR = 1.26, 95% CI 1.04–1.53, *p* = 0.019). Multivariate analysis incorporating age, sex, and tumor stage (Fig. 4C, D) confirmed independent prognostic associations in SKCM (adjusted HR = 0.756, 95% CI 0.650–0.879, *p* < 0.001) and KIRC (adjusted HR = 0.790, 95% CI 0.682–0.914, *p* = 0.002), with directional consistency maintained across 10 of 11 cancer types. In contrast, the stress-metabolic TA-HEV score showed statistically significant adverse effects in STAD (HR = 1.19, 95% CI 1.02–1.41, *p* = 0.028) and KIRC (HR = 1.30, 95% CI 1.11–1.53, *p* = 0.001) in univariate analysis (Fig. 4A, B). Multivariate analysis (Fig. 4C, D) confirmed independent adverse associations in KIRC (adjusted HR = 1.272, 95% CI 1.084–1.493, *p* = 0.003) and ESCA (adjusted HR = 1.410, 95% CI 1.057–1.881, *p* = 0.020), with directional consistency across 9 of 11 cancer types. Although several cancer types did not reach nominal significance, effect estimates were directionally adverse in most cohorts, indicating a consistent trend toward poorer survival associated with the stress-metabolic state (detailed HRs, 95% CIs and p-values in Supplementary Table S5).

Stratification of patients by high or low inflammatory and stress-metabolic TA-HEV scores revealed distinct survival patterns (Fig. 4C). In SKCM, patients with high inflammatory and low stress-metabolic scores exhibited significantly improved survival, whereas those with high stress-metabolic and low inflammatory scores showed poor outcomes. The high inflammatory TA-HEV group also exhibited an overall increase in estimated immune cell fractions, with particularly prominent enrichment of CD8⁺ T cells (Fig. 4D). In contrast, prognosis in lung squamous cell carcinoma (LUSC) was primarily driven by the stress-metabolic TA-HEV score, and a high inflammatory TA-HEV score was not accompanied by a clear increase in estimated immune cell fractions (Fig. 4E, F).

Collectively, these results define TA-HEVs as a transcriptionally and functionally coherent endothelial population.

## Discussion

TA-HEVs are often described as beneficial gateways for lymphocyte entry into tumors. However, comprehensive analyses of their functional roles across cancers remain limited. Here, by integrating single-cell and bulk transcriptomic data from multiple cancer types, we delineate two coherent TA-HEV programs–an inflammatory state and a stress-metabolic state–that exhibit opposing, tumor-context-dependent associations with patient outcomes.

To identify TA-HEVs, we constrained subclustering to the tumor-associated venous endothelial lineage. According to the classical definition, HEVs are specialized post-capillary venules. Thus, our analytic strategy is consistent with the developmental and anatomical identity of TA-HEVs [11, 12]. Beyond canonical HEV marker expression, we also applied complementary criteria, including signature scores, pathway enrichment, and a pseudotime analysis. This biologically coherent and dataset-agnostic framework suggests that the observed TA-HEV states reflect genuine biological change rather than artifacts. Accordingly, the cancer-specific differences in TA-HEV prevalence and state usage can be interpreted as a valid, tumor-context-dependent heterogeneity.

The inflammatory TA-HEV program encompasses innate immune stimulation, cytokine/chemokine signaling, and MHC-II antigen presentation. In SKCM, the inflammatory TA-HEV state coincides with higher immune cell infiltration, most prominently CD8⁺ T-cells, and is associated with improved survival. These observations are consistent with reports that TA-HEVs are associated with enhanced lymphocyte recruitment, correlating with clinical benefit [13]. In BRCA, STAD, and LUSC, a high inflammatory TA-HEV score was not accompanied by a clear increase in estimated immune cell fractions and was associated with adverse prognosis. These findings suggest that similar inflammatory transcriptional programs may not translate into effective immune infiltration within immunosuppressive microenvironments [14]. The stress-metabolic TA-HEV program characterized by the unfolded protein response (UPR), heat shock pathways, oxidative phosphorylation, and ATP biosynthesis is associated with poor outcomes across multiple cancers. This observation aligns with literature suggesting endothelial metabolic reprogramming and ER stress/UPR pathways can be associated with immunosuppressive vascular features and reduced therapy response [15]. Overall, TA-HEV function is not uniform but varies by state and tumor context. Although the inflammatory and stress-metabolic programs described here share features with previously reported endothelial activation and stress-response states, they were identified within a HEV-defined venous endothelial compartment. We therefore interpret them not as generic endothelial programs, but as HEV-resolved functional states that retain core HEV identity.

Our results also underscore the clinical significance of TA-HEV state subtype. Collapsing TA-HEV subclusters into state-specific TA-HEV scores provides a practical, cohort-scale means of risk stratification in cancer patients. In immunogenic tumors such as SKCM, inflammatory-high/stress-metabolic-low patients showed more favorable survivals, whereas a higher stress-metabolic TA-HEV score consistently marked high-risk patients across tumor contexts. These findings further suggest that vascular-targeted or HEV-modulating strategies should be context-tailored. These observations suggest that TA-HEV states may inform context-dependent vascular or endothelial-focused strategies, particularly in settings where stress-metabolic programs are associated with adverse immune landscape and microenvironments that may be less conducive to effective antitumor immunity.

Although scRNA-seq data limit sample numbers, we mitigated this by pan-cancer integration and by validating single-cell findings with bulk RNA-seq deconvolution. Deconvolution cannot reproduce single-cell resolution, but it offers cohort-scale readout. To our knowledge, this is the first pan-cancer definition of TA-HEV states with prognostic relevance.

Several limitations of the present study warrant consideration. The transcriptional characterization of the stress-metabolic TA-HEV subgroup is based on scRNA-seq data, which carries an inherent risk of dissociation-induced artifact. To address this, we compared the top 20 marker genes of each TA-HEV subgroup against the dissociation-induced gene reference set published by van den Brink et al. [16], and calculated artifact module scores across TA-HEV cells. The scores were similarly low across both subgroups (Fig. S5). These results suggest that the identified transcriptional programs appear to be reflective of biological states. Validation using snRNA-seq would provide additional confidence.

Additionally, the prognostic significance of TA-HEV subtype signatures was validated using TCGA bulk RNA-seq data, which is currently the most comprehensive resource for pan-cancer clinical outcome analysis at this scale. Cell-type specificity analysis supported the endothelial enrichment of both TA-HEV signatures across major tumor-resident cell types, though the degree of specificity varied between subprograms. These findings do not alter the overall prognostic associations of the TA-HEV states identified in this study, and the bulk-based validation approach remains appropriate given the current availability of pan-cancer clinical outcome data.

TA-HEV states differ transcriptionally and functionally, vary by tumor context, and are associated with opposing survival outcomes. These results propose TA-HEV states as potential biomarkers and therapeutic targets and highlight their context-dependent dual roles.

### Methods

#### Single-cell RNA-seq data collection and preprocessing

scRNA-seq data from 317 primary tumor tissue samples (307 patients) across 11 solid cancer types were analyzed, totaling ~ 1.3 million cells after quality control. The cohorts included: stomach adenocarcinoma (31 patients, total 140,217 cells); breast invasive carcinoma (52 patients, total 347,945 cells); colorectal cancer (29 patients, total 73,777 cells); esophageal carcinoma (60 patients, total 192,078 cells); thyroid carcinoma (7 patients, total 65,580 cells); non-small cell lung cancer (50 patients, total 105,435 cells); melanoma (10 patients, total 132,464 cells); cutaneous squamous cell carcinoma (10 patients, total 26,299 cells); pancreatic adenocarcinoma (16 patients, total 42,272 cells); head and neck squamous cell carcinoma (21 patients, total 100,499 cells); and renal cell carcinoma (8 patients, total 100,916 cells). Only datasets without prior cell-type enrichment were included, while peripheral blood, metastatic, matched normal, and healthy donor samples were excluded. This study focused exclusively on tumor-associated endothelial cells, as HEVs are not present under homeostatic conditions in non-lymphoid peripheral tissues and emerge specifically in pathological contexts. The molecular characterization of normal tissue endothelial cells was therefore outside the scope of the present study. Detailed information for each dataset is provided in Supplementary Table S6.

Raw gene count matrices were processed using the Seurat R package [17]. Cells with < 200 detected genes, > 20% mitochondrial reads, or identified as doublets by DoubletFinder were excluded [18]. Gene counts were normalized with *NormalizeData()* (scale factor = 10,000), highly variable genes (HVGs) were identified using *FindVariableFeatures()*, and data were scaled with *ScaleData()*. Dimensionality reduction was performed by principal component analysis (PCA) with *RunPCA()*. A shared nearest-neighbor graph was constructed with *FindNeighbors()*, followed by clustering using *FindClusters()* with a resolution parameter of 0.8. Uniform manifold approximation and projection (UMAP) was performed with *RunUMAP()*, with dataset-specific parameter settings for the number of principal components (dims = 10–20). Unless otherwise specified, default parameters of the Seurat functions were applied.

### Data integration and unsupervised clustering

ECs were characterized from the total cells of each dataset based on the expression of pan-EC markers (PECAM1, VWF, CDH5, and PLVAP). Count matrices of the identified ECs were then integrated and processed as described above, including normalization, identification of HVGs, scaling, and dimensionality reduction by PCA. Batch correction was performed using the Harmony R package with dims.use = 20 parameter [19].

For visualization, *RunUMAP()* and *FindNeighbors()* were applied with reduction = “harmony” and dims = 20. Unsupervised clustering was performed using *FindClusters()* with a resolution of 0.8. Major EC types were annotated according to well-established markers, and clusters expressing signatures of non-endothelial cell types were excluded, after which the above steps were repeated.

The venous endothelial subgroup was further reclustered at multiple resolutions, and cluster purity was evaluated using the ratio of global unshifted entropy (ROGUE) value [20]. The resolution yielding the highest ROGUE score (resolution = 1.35) was selected and applied in *FindClusters()*. TA-HEV subclusters were identified based on the enrichment of a previously reported 34-gene HEV signature [21, 22]. The HEV signature score was calculated using *AddModuleScore()* in Seurat.

### Differential expression, trajectory, and functional analysis

Cluster-specific DEGs were identified with Seurat *FindAllMarkers()* (adjusted *p* < 0.05). Gene Ontology (GO) enrichment was performed with clusterProfiler [23]. Differentiation states were inferred by CytoTRACE2 [24], and pseudotime trajectories constructed using Monocle3 [25]. Functional module scores, including MHC class II (MHC-II, *KEGG NETWORK: N00590*), Antigen processing (*GO:0019886*), Response to heat stress (*R-HSA-3371556*), Response to unfolded protein (*GO:0034620*), Oxidative phosphorylation (*HALLMARK_OXIDATIVE_PHOSPHORYLATION*), and ATP metabolic process (*GO:0046034*) were calculated with Seurat *AddModuleScore(). *The gene lists used for signature score calculation, including HEV signature genes, were provided in Supplementary Table S9. To assess potential dissociation-induced artifact in the identified TA-HEV subgroups, the top 20 marker genes of each subgroup (inflammatory: subclusters 0, 1, and 12; stress-metabolic: subclusters 5 and 6) were compared against the published dissociation-induced gene list [16], and artifact module scores were calculated across all TA-HEV cells; results are provided in Fig. S5 and Supplementary Table S11.

### Bulk RNA-seq data validation

Bulk RNA-seq data from TCGA pan-cancer cohort were analyzed by single-sample gene set enrichment analysis (ssGSEA) using the top 20 upregulated DEGs from each TA-HEV subcluster. Inflammatory (subclusters 0, 1, 12) and stress-metabolic (subclusters 5, 6) TA-HEV scores were derived as the averages of ssGSEA scores from their respective subclusters. For survival analysis, these scores were standardized by Z-score transformation within each cancer type and analyzed by univariate Cox proportional hazards regression for overall survival (OS), censored at 10 years. Kaplan–Meier (KM) curves were generated after stratifying patients into four groups by the median values of both scores, and survival differences were tested using the log-rank method. To assess the independent prognostic value of TA-HEV scores, multivariate Cox proportional hazards regression was additionally performed incorporating age, sex, and stage as covariates. Cell-type specificity of TA-HEV subcluster-specific signatures was assessed by computing average expression scores for HEV across major tumor-resident cell types in publicly available scRNA-seq datasets (GSE131907, GSE132465, GSE144236, GSE148071, GSE155698, GSE161529, GSE167297, GSE172577, GSE178481, GSE183904, GSE184362, GSE188711, GSE277165); results are provided in Fig.S6.

### Data visualization and statistical analysis

Gene expression heatmaps, dot plots, and box plots were generated using the SeuratExtend package, whereas UMAP plots were produced with Seurat. GO heatmaps were created with ComplexHeatmap, and bar plots as well as survival analysis plots were generated using ggplot2. Statistical analyses were performed using R 4.2.2. Statistical tests included Kruskal–Wallis, Wilcoxon, *t*-test, and log-rank tests, with *p* < 0.05 considered significant. HRs and 95% confidence intervals (CIs) were estimated using Cox proportional hazards models. Immune cell composition in TCGA samples was inferred using CIBERSORTx with the LM22 signature matrix, yielding estimated immune cell fractions for each sample [26]. All software and tools used in this study are summarized in Supplementary Table S8.

## Supplementary Information

Below is the link to the electronic supplementary material.

Supplementary Material 1.

Supplementary Material 2.

Supplementary Material 3.

Supplementary Material 4.

Supplementary Material 5.

Supplementary Material 6.

Supplementary Material 7.

Supplementary Material 8.

Supplementary Material 9.

Supplementary Material 10.

Supplementary Material 11.

Supplementary Material 12.

## Data Availability

Publicly available scRNA-seq datasets used in this study are available from the Gene Expression Omnibus (GEO). Accessions for datasets used in this study: STAD: GSE183904, GSE176078, BRCA: GSE176078, GSE161529, CRC: GSE132465, GSE188711, ESCA: GSE160269, THCA: GSE184362, NSCLC: GSE148071, GSE131907, MELA: GSE277165, cSCC: GSE144236, PAAD: GSE155698, HNSC: GSE164690, GSE172577, RCC: GSE178481. Bulk RNA-seq data from The Cancer Genome Atlas (TCGA) Pan-Cancer Atlas cohort were obtained from the Genomic Data Commons (gdc.cancer.gov/about-data/publications/pancanatlas), including the following TCGA cohorts: TCGA-BRCA, TCGA-COAD, TCGA-ESCA, TCGA-HNSC, TCGA-KIRC, TCGA-LUAD, TCGA-LUSC, TCGA-PAAD, TCGA-SKCM, TCGA-STAD, and TCGA-THCA.
