# Supplementary material for: Transcriptional heterogeneity of tumor-associated high endothelial venules defines inflammatory and stress-metabolic states with distinct prognostic associations
Source: Discov Oncol. 2026 May 8;17:964. doi: 10.1007/s12672-026-05162-2 (PMC13323434; doi:10.1007/s12672-026-05162-2)
Supplement: Supplementary file 11 — Supplementary Material 11. [file 12672_2026_5162_MOESM11_ESM.pdf]

Supplementary Figures

Supplementary Figure 1. Distribution of endothelial cell (EC) subtypes within tumor-associated endothelial cells.

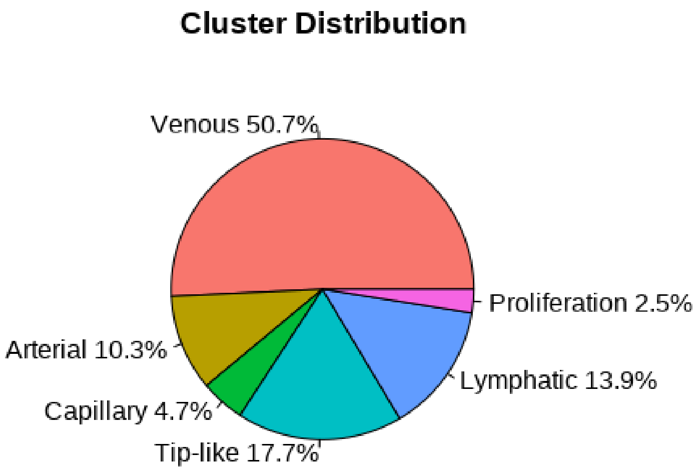

Supplementary Figure 2. UMAP visualization shows even distribution of venous EC subclusters across tumor types and datasets.

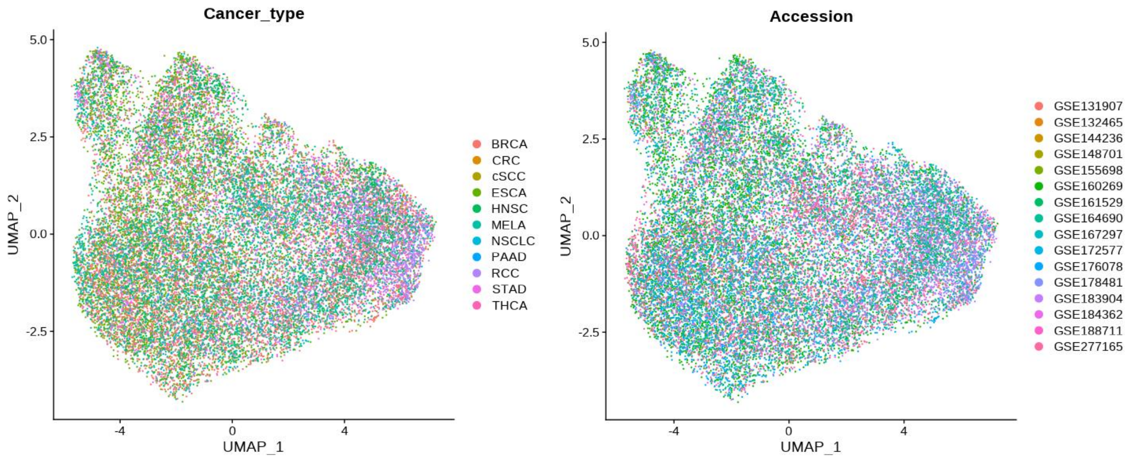

Supplementary Figure 3. UMAP visualization of venous EC subclusters classified as tumor-associated high endothelial venule (TA-HEV), immature, and mature venous ECs (VECs).

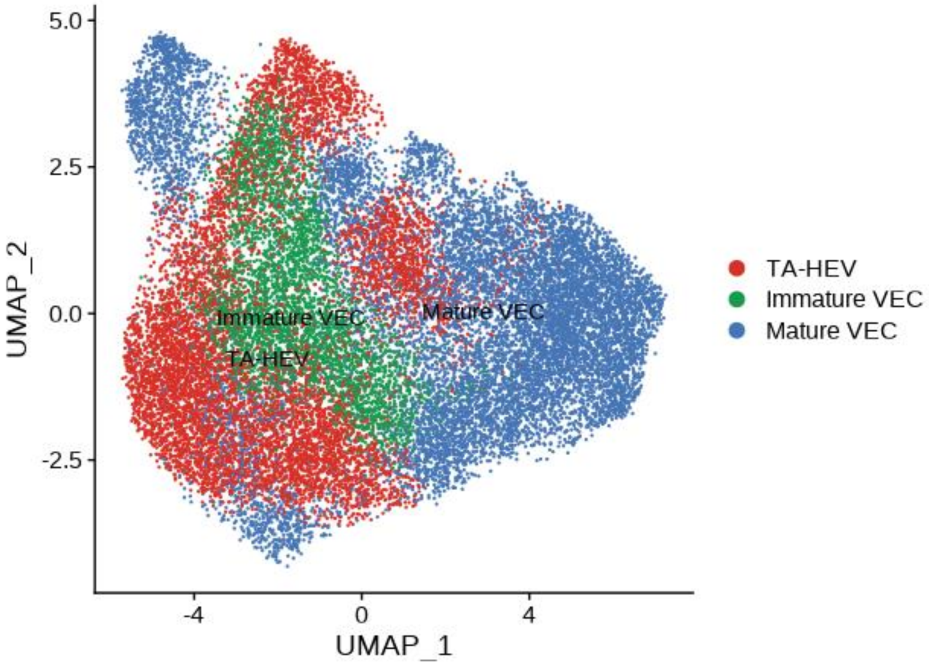

Supplementary Figure 4. Subcluster 0 shows significantly higher IL6 expression among TA-HEV subclusters. (Wilcoxon rank sum test, $*p < 0.05$, $**p < 0.01$, $***p < 0.001$, $****p < 0.0001$)

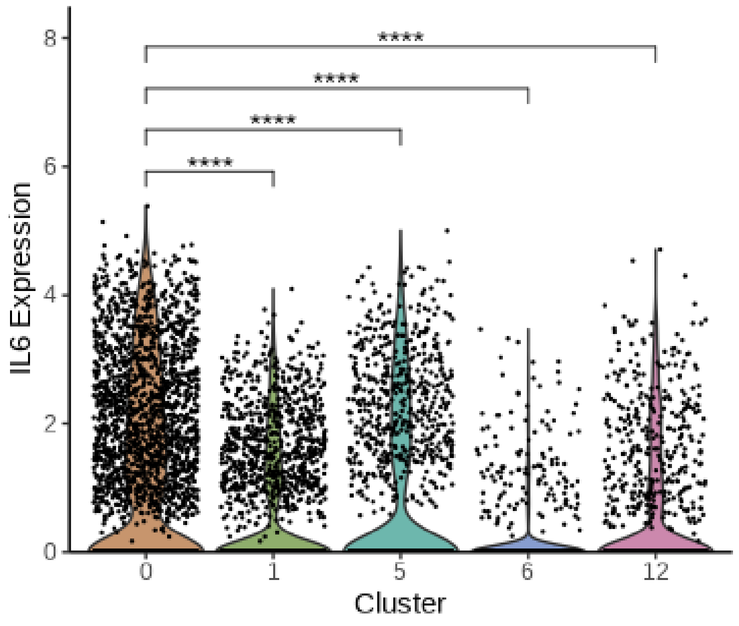

Supplementary Figure 5. Distribution of dissociation artifact scores across TA-HEV subgroups.

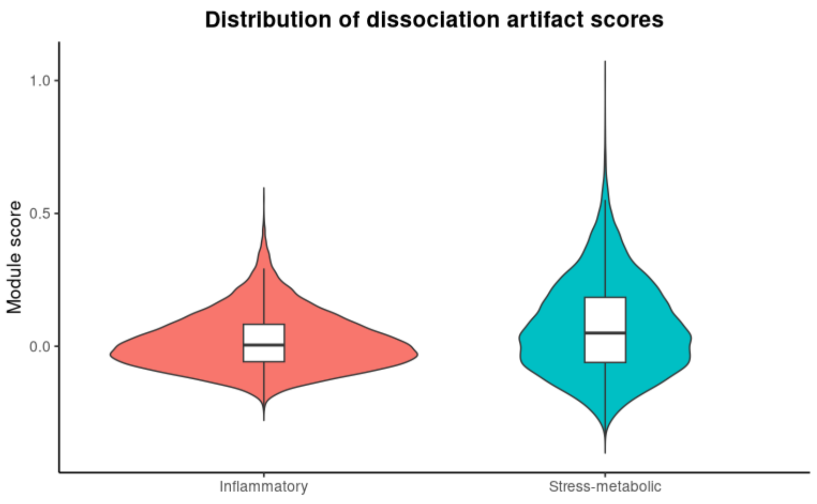

Supplementary Figure 6. Supplementary Figure X. Cell-type distribution of TA-HEV signature expression across tumor microenvironment cell types.

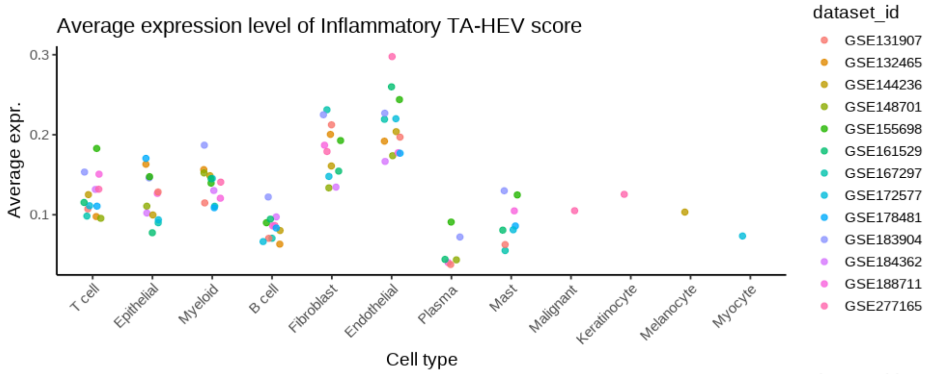
Average expression level of Inflammatory TA-HEV score
Average expr.
0.3
0.2
0.1
T cell
Epithelial
Myeloid
B cell
Fibroblast
Endothelial
Plasma
Mast
Malignant
Keratinocyte
Melanocyte
Myocyte
Cell type
dataset_id
GSE131907
GSE132465
GSE144236
GSE148701
GSE155698
GSE161529
GSE167297
GSE172577
GSE178481
GSE183904
GSE184362
GSE188711
GSE277165

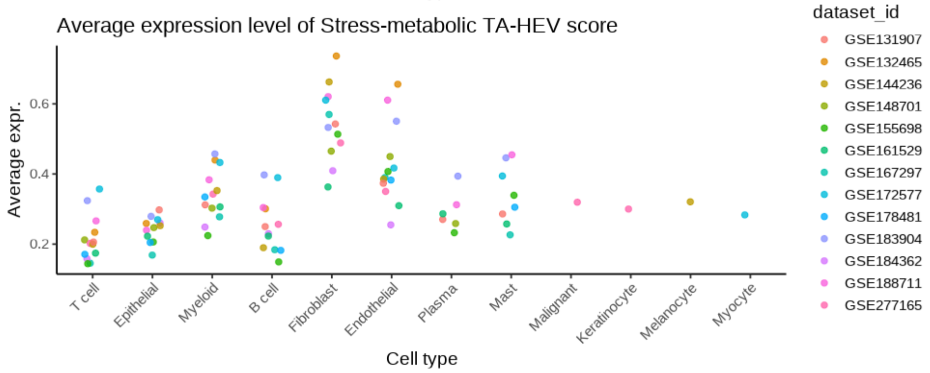
Average expression level of Stress-metabolic TA-HEV score
Average expr.
0.6
0.4
0.2
T cell
Epithelial
Myeloid
B cell
Fibroblast
Endothelial
Plasma
Mast
Malignant
Keratinocyte
Melanocyte
Myocyte
Cell type
dataset_id
GSE131907
GSE132465
GSE144236
GSE148701
GSE155698
GSE161529
GSE167297
GSE172577
GSE178481
GSE183904
GSE184362
GSE188711
GSE277165
